# Clinical study of bilateral decompression via vertebral lamina fenestration for lumbar interbody fusion in the treatment of lower lumbar instability

**DOI:** 10.3892/etm.2013.903

**Published:** 2013-01-17

**Authors:** SHUGUANG GUO, JUNYING SUN, GENLIN TANG

**Affiliations:** 1 Orthopedic Department, The First Affiliated Hospital of Soochow University, Jiangsu, Suzhou 215006;; 2 Orthopedic Department, The People’s Hospital of Taizhou in Jiangsu Province, Jiangsu, Taizhou 225300, P.R. China

**Keywords:** vertebral lamina fenestration, threaded fusion cage, interbody fusion, lower lumbar instability

## Abstract

The aim of this study was to observe the clinical effects of bilateral decompression via vertebral lamina fenestration for lumbar interbody fusion in the treatment of lower lumbar instability. The 48 patients comprised 27 males and 21 females, aged 47–72 years. Three cases had first and second degree lumbar spondylolisthesis and all received bilateral vertebral lamina fenestration for posterior lumbar interbody fusion (PLIF) using a threaded fusion cage (TFC), which maintains the three-column spinal stability. Attention was given to ensure the correct pre-operative fenestration, complete decompression and the prevention of adhesions. After an average follow-up of 26.4 months, the one year post-operative X-ray radiographs suggested that the successful fusion rate was 88.1%, and this was 100% in the two-year post-operative radiographs. Moreover, the functional recovery rate was 97.9%. Bilateral vertebral lamina fenestration for lumbar interbody fusion is an ideal surgical method for the treatment of lower lumbar instability. The surgical method retains the spinal posterior column and middle column and results in full decompression and reliable fusion by a limited yet effective surgical approach.

## Introduction

Among the studies on chronic lumbosacral pain (1), lower lumbar instability (2) has attracted much clinical attention. Since Scheufler *et al*(3) reported that segmental instability is caused by the degeneration of the fibrous ring-nucleus pulposus complex in 1944, the diagnostic criteria for lower lumbar instability has been more consistently defined (4) as the appearance of anomalies in the lower lumbar spine (L_3_-S_1_) and corresponding clinical symptoms and imaging changes in the motion segment under a normal physiological workload. Lower lumbar instability is clinically characterized by the following: lower back pain with hip and lower limb referred pain; sudden pain in the waist due to changes in position; stiff and blocked stair-stepping or excessive activity in the spinous process when standing; intermittent claudication; myasthenia in the lower limbs, and defecation and urination obstructions in seriously affected patients. The comparison of lumbar dynamic position radiographs in hyperextension and hyper-flexion positions is an important factor in diagnostic imaging. Lumbar X-ray radiographs in hyperextension and hyperflexion positions suggest that the L_3–5_ segments slide forward and back by >3 mm and the L_5-_S_1_ segments slide by >4–5 mm. The angle changes of the endplate are >10°, suggesting lower lumbar instability (5). den Boer *et al*(6) identified that lumbar instability and lumbar segmental degeneration are characterized by abnormal stress distribution of the nuclear-fiber ring, loose column ligaments and repeated sprains, which explains the pathogenesis of the usually merged lumbar intervertebral disc herniation. The authors also highlighted the clinical significance of lumbar mechanism dysfunction of the three columns on the pathogenesis of lumbar degenerative instability and lower lumbar instability in iatrogenic surgery and emphasized the necessity of maintaining the stability of the three columns in treatment. Another study (7) reported that the surgical approach for conventional posterior lumbar decompression and fusion is typically omniposterior resection. Intra-operative vertebral laminae, spinous processes and other important posterior column structures should receive wide excision. However, the view of decompression in minimally invasive surgery (8) is less clear than that in open surgery and has a number of disadvantages, including incomplete spinal decompression, potential vice injury risk, a steep learning curve and the requirement for special equipment. Extensive experience of open surgery is also required. All these factors are unfavorable for the clinical application of minimally invasive surgery. A total of 48 patients with lower lumbar instability received posterior lumbar interbody fusion (PLIF) via bilateral vertebral lamina fenestration using a threaded fusion cage (TFC) from 2006 to 2009. The surgery retained the spinous process and posterior longitudinal ligament. It is a practical procedure with a good curative effect and indicates that surgical methods for lower lumbar instability may simultaneously achieve the important clinical aims of strong fusion, complete decompression and stability maintenance of the three columns.

## Materials and methods

### General data

Among the 48 patients, 27 were males and 21 were females, aged 47–72 years, with an average age of 62.3 years. These patients had disease durations from 3 months to 15 years, with an average of 2.6 years. All cases had varying degrees of chronic lumbosacral pain which was aggravated after standing or walking for a long time, and was not relieved following conservative clinical treatment. The study included 25 cases of intermittent claudication, 36 cases of unilateral lower limb pain and reduced muscle strength, 8 cases of bilateral lower limb pain and reduced muscle strength, 32 cases of restricted lower limb tendinous reflex and 1 case of defecation and urination obstructions. This study was conducted in accordance with the Declaration of Helsinki and with approval from the Ethics Committee and the Institutional Review Board (IRB) of the First Affiliated Hospital of Soochow University. Written informed consent was obtained from all participants.

### Imaging diagnosis

**Figure 1.**
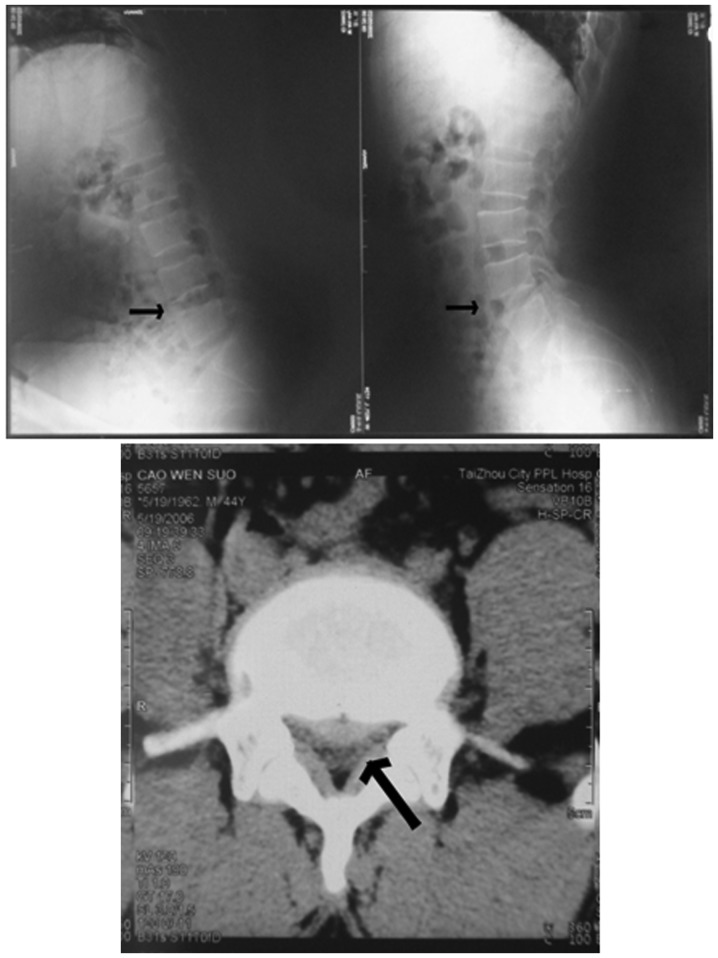
Male, 53-year-old patient with chronic lumbosacral pain for >5 years. Preoperative lumbar dynamic topography (black arrow) suggested vertebral instability between waist 4 and waist 5. Lumbar computed tomography (CT; black arrow) suggested a prolapsed intervertebral disc between waist 4 and waist 5, which clearly compressed the dural sac.

All patients received lumbar spine X-ray radiographs (in the normotopia and lateral positions), computed tomography (CT) and magnetic resonance imaging (MRI) examination. The results indicated that the 48 cases of lumbar disc herniation combined with intervertebral lesion were all suppressed by the dural sac and nerve root. Of these, there were 35 cases of single-level lesions, 9 cases of double-level lesions and 4 cases of triple-level lesions. The side with central-type disc herniation and intervertebral lesion was in the right sternum in 46 patients and 19 patients had partial lateral central-type disc herniation. Furthermore, 32 cases of lumbar degeneration, 27 cases of small joint hyperplasia, 8 cases of longitudinal ligament ossification, 1 case of disconnected vertebral arch, 2 cases of spondylolisthesis I and 1 case of spondylolisthesis II were observed. Comparison of lumbar dynamic position radiographs of L_3-_S_1_ in the hyperextension and hyperflexion position demonstrates that the inferior margin shift of the adjacent vertebra of the intervertebral lesion was >4 mm and the angle change between the adjacent inferior margin of the inferior vertebra and the superior margin of the inferior vertebra was >10°. The intervertebral lesion was consistent with the nerve compression (Fig. 1) and was diagnosed as lower lumbar instability type I. The intervertebral lesion characterized by type I, combined with lumbar spondylolisthesis I–II, was diagnosed as type II. Among the cases in this study, 45 patients were diagnosed as type I and 3 patients as type II.

### Surgical method

**Figure 2.**
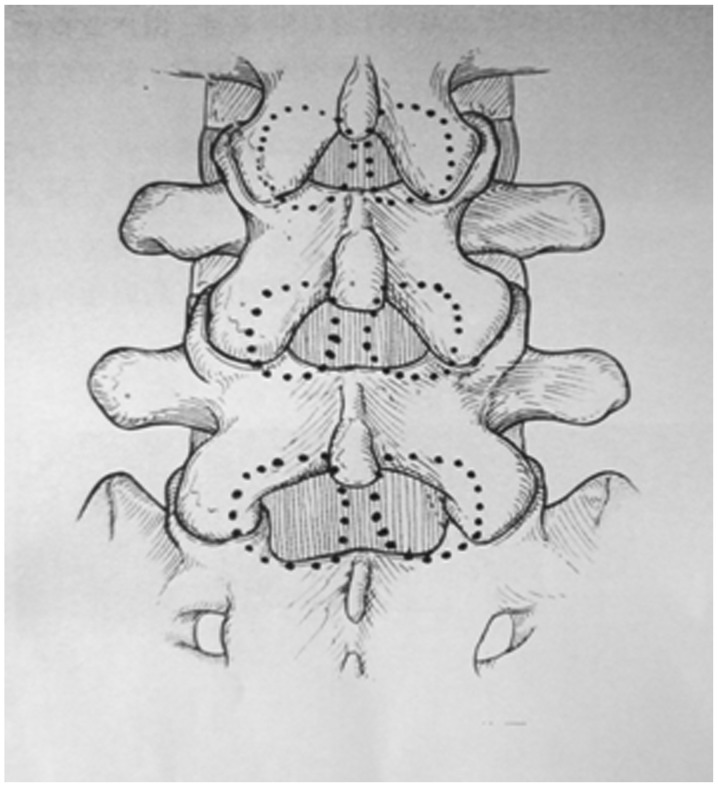
Black dotted line demonstrates the range of bilateral vertebral lamina fenestration for lumbar interbody fusion.

Patients received lumbar anesthesia or general anesthesia in the prone position and conventional sterile surgery, with padded thorax and abdomen. The intervertebral lesion was exposed in a median incision of the posterior lumbar. According to the pre-operative CT and MRI, the herniated disk side was operated on first. Following the removal of the 1/2 to 1/3 of the interior small joint, adjacent lamina margin and its ligamentum flavum, undercutting decompression was performed in the nerve root canal and spinous basal parts. Ligamentum flavum in the lateral spinal canal was carefully removed to form the bone window with a diameter of 14–18 mm (Fig. 2). After the tensions of the nerve root and dural sac were significantly reduced, the intervertebral lesion was protected and led to the middle. Following this, the fiber ring was cut and excision of the nucleus pulposus was performed. The other side underwent the same surgery. The spinal canal was flushed with ice-cold normal saline (NS). Compression factors such as osteophytes of vertebrae posterior margin, longitudinal ligament after ossification and ligamentum flavum in small joint were detected and removed, to avoid damage to epidural venous plexus. The TFC surgery protection sleeve was placed on one side of the bone window. Under the guidance of a C-arm X-ray machine, the superior and inferior cartilage boards of the clearance were removed with a special cutter. A TFC filled with crushed bones was implanted into a suitable location, with a diameter of 10–14 mm and a length of 20–25 mm. The posterior margin was usually located at 3–5 mm before the posterior margin of the vertebrae. The bones may be reconditioned by the crushed bones in the margins of the small joints, vertebral lamina and spinous processes cut off during decompression. The other side underwent the same surgery. The TFC in each side was attended to, to ensure that it avoided crossing the center line of the clearance. Another ice-cold NS flushing and spinal canal detection were carried out to remove the residual cartilage fragments and nucleus pulposus. A poly(d,l-lactic acid) absorbable anti-adhesive membrane was used to cover the bone window and close the incision. One drainage tube was placed in each side of the incision and removed after 48–72 h, depending on the lead flow. The pedicle screw and connecting longitudinal stem of the bilateral vertebra of the lesion clearance were placed first in type II patients. The reduction of spondylolisthesis, as well as appropriate lesion clearance and fixation, was followed by decompression via vertebral lamina fenestration for TFC fusion.

## Results

### Bridwell method evaluation

**Figure 3.**
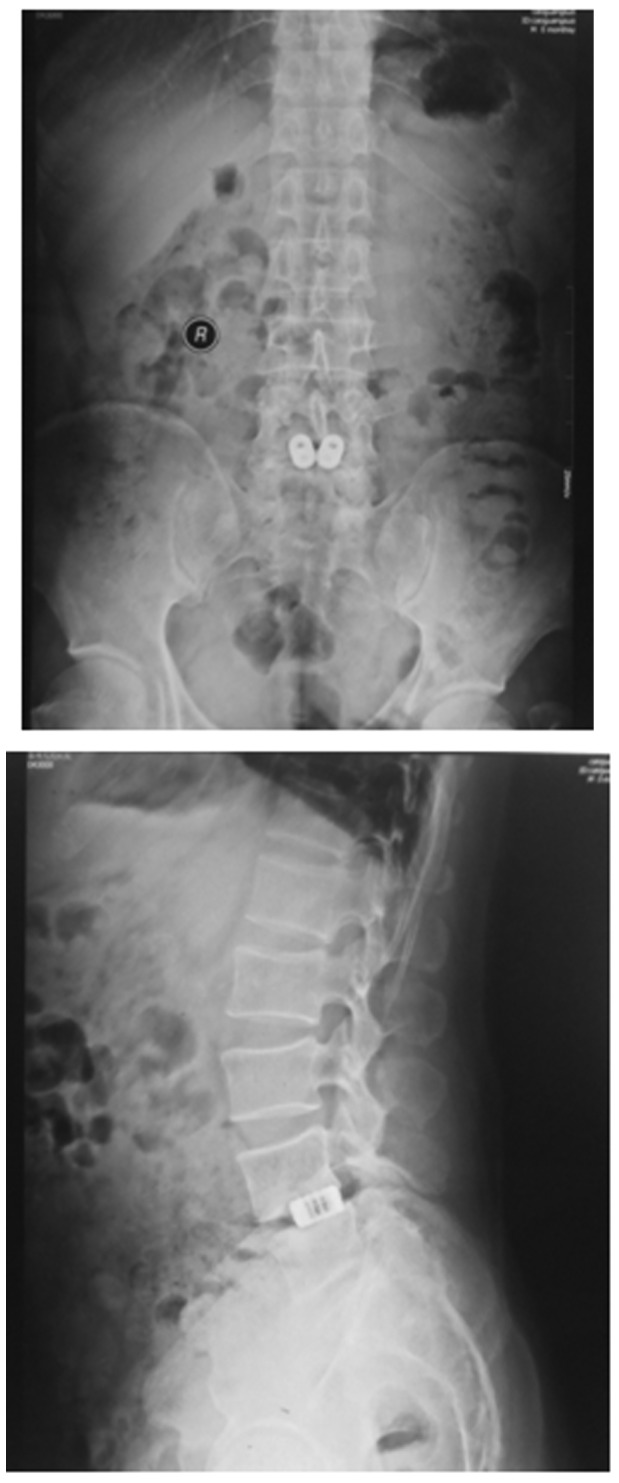
Images captured following bilateral vertebral lamina fenestration for lumbar interbody fusion.

All patients received follow-up for a period of 20 months to 3 years, with an average of 26.4 months. The method created by Bridwell *et al*(9) was used to evaluate the lumbar fusion conditions. The X-ray radiographs one year after surgery suggested that the fusion rate between intervertebral grade I and II was 88.1%. At two years post-surgery, the fusion rate between intervertebral grades I and II was 100% (Fig. 3),. Two cases of mildly reduced intervertebral space height were observed; however, the reduction was <10%.

### Macnab curative effect

Functional recovery is the curative effects evaluated by the improved Macnab criteria, as follows. Optimal effects: disappearance of pain, no motor dysfunction, and a return to work and activities; good effects: occasional pain, disappearance of main symptoms, normal muscle strength, a negative result in the straight legs raising test and femoral nerve stretch test, light physical labor is tolerable; passable effects: symptoms are improved but pain persists, and patients are capable of looking after themselves in their daily life although unable to work; poor effects: patients suffer nerve compression and require further surgery. Using these criteria, in our study a total of 42 cases (87.5%) were optimal, 5 cases (10.4%) were good and 1 case (2.0%) was passable. All cases exhibited relieved lower limb pain and the back pain relief rate was 97.9%. One patient demonstrated mild cerebrospinal leakage in the incision 7 days after surgery; this healed well after local processing and the stitches were removed after 3 weeks. One type II case exhibited repeated left waist pain from six months after surgery. The 14 month post-operative X-ray radiographs suggested good interbody fusion; however, the pedicle screw system was loosened and the back pain was relieved following its removal.

## Discussion

Lower lumbar instability is usually accompanied by lumbar disc herniation, lumbar spondylolisthesis and segmental lumbar spinal stenosis. With the exception of a few cases with mild symptoms and short attacks who received conservative treatments for short-term relief, all cases suffered repeated attacks and curing the disease was difficult. These patients demonstrated clear surgical indications and were suitable candidates for lumbar fusion (10). This study aimed to achieve lower lumbar fusion stability and spinal canal decompression, eliminate nerve compression syndrome, restore the normal spinal mechanism of balance, preserve the spinal physiological function and delay the degenerative trend of the adjacent normal lumbar segments. Particularly in those aged >35 years with lumbar disc herniation, the abnormal activities of motion segments indicated by lumbar dynamic position radiographs should be considered as absolute indications of lumbar fusion during nucleus pulposus enucleation. Otherwise post-operative lumbar spinal instability may be aggravated. The three columns of the spine should be kept stable for spinal decompression. Ray (11) reported 208 cases of PLIF using TFC with a three year follow-up and identified a bone fusion rate of 96%. The author indicated that vertebral fusion using TFC is based on full decompression of the lumbar spinal canal. This is characterized by the inclusion of a large number of micropores and locally implanted, autologous cancellous bone providing a large fusion area, as well as equal and partial loading of the spinal load, reducing axial shear. As a result, PLIF using TFC has become a common surgical procedure in the treatment of lumbar instability, due to the improved fusion effect compared with that of traditional lumbar fusion (12).

In observational studies of PLIF using TFC and other inter-body fusion cages in the treatment of lumbar instability (13), several scholars identified that the vertebral fusion rate and rate of clinical symptom improvement are variable, with possible adverse complications, including loose or slipped fusion cages, fusion failure and aggravated lumbar and spinal instability. Several clinical observations (14) suggested that these adverse complications may be related to the different approaches used in implanting fusion cages with spinal decompression. A clinical study (15) demonstrated that patients with lower lumbar spinal instability should preferentially receive fusion and stability surgery. However, patients are often admitted with long-term degeneration of stability and significant spinal stenosis. Therefore, they should receive spinal canal decompression surgery at the same time. The strong fusion and complete decompression in the treatment of lumbar spinal instability and surgical decompression, which maintains spinal stability, should be used if possible (16), particularly in the protection or persistence of the posterior column. Furthermore, the ‘limited surgery approach’ should be advocated to reduce the iatrogenic instability factors and provide the best anatomical and biomechanical foundation for effective vertebral interbody fusion. Martin *et al*(17) reported that, the spinal decompression and implantation in PLIF were mainly used in laminectomy or hemilaminectomy, with postoperative apparently damaged stability of central and posterior column and exposed dural sac. The external surface of the latter is vulnerable to adhesion compression of the erector spinae and dural fiber scarring following surgery (18). Iatrogenic injury of the lumbar spinal stability mechanism, as well as the damaged anterior fiber ring and posterior longitudinal ligament, which increase the tendency of spinal pivoting and slipping, should be regarded as important signs of post-operative poor fusion and incompletely improved symptoms. Bilateral decompression via vertebral lamina fenestration for TFC fusion was combined with the principle of minimizing damage using minimally invasive surgery (19) to further retain the three column frame of the spine. This fulfils the limited surgery approach, as well as thorough spinal decompression and effective vertebral interbody fusion.

A related study (20) demonstrated that the clearance of the L_3–5_ bilateral articular processes of an adult are on average 33.5, 40.1 and 47.4 mm, respectively. With the addition of a conventional resection of 1/3-1/2 inside the small joint for intra-operative decompression, the diameter of the general bone window is 14–18 mm and the area is 2.4–3.0 mm^2^. The full spinal undercutting decompression (21) requires careful surgery. The proper usage of TFC input protection devices is able to perfectly resolve the contradiction between the ‘limited approach’ and the exposed operative field to avoid nerve damage, iatrogenic instability and other adverse complications. The treatment requires the following: i) surgical personnel should coordinate with each other and strictly abide by the surgical programs step by step. The surgeon must have a good understanding of the lumbar spinal anatomical structure and have considerable experience of lumbar spine surgery. Intra-operatively, a good operative field should be exposed and in each step the regional anatomy of the lumbar spine should be considered (22). The adverse complications of nerve injury may be avoided through accurate and careful surgery, as well as coordination during surgery. Our study had 1 case of post-operative mild cerebrospinal leakage, which was related to spinal adhesion caused by serious compression and repeated loosening, separation and complete removal of epidural band cicatrix, without nervous dysfunction following post-operative controversial treatment. ii) Accurate fenestration and full decompression are the keys to successful surgery. During fenestration, the position and direction of the vertebral clearance, as well as the correlation between vertebral clearance and the laminal clearance should be given attention. The window formed was quasi-circular, with a larger transverse diameter, beneficial for the placement of the nerve hook and TFC protection devices. Part of the cortex in the basilar section of the spinous process may be removed to allow fenestration. Following fenestration, full undercutting decompression should be performed, with equal attention given to the central spinal canal and nerve root canal, particularly for the removal of pressure factors, including ossified posterior longitudinal ligament, hyperplastic osteophytes in the posterior vertebrae, medial margin of cohered and hyperplastic articular process, narrow parapsidal furrow of the superior articular process and a hyposulculus in the pedicle of the vertebral arch, lateral recess and lateral ligament flavum (21). While removing the osseous oppression, the soft pressure factors of the nucleus pulposus, the organized, calcified or hypertrophic fibers of the ligament fiavum and the posterior longitudinal ligament should not be ignored. During the decomposition of the adhesion, the integrity of the dural sac should be maintained. The spinal canal should be checked again after placing the TFC, removing iatrogenic pressure factors, including fiber ring debris of the nucleus pulposus and crushed bones from the resection of the lamina. iii) Correct understanding of the placing of the TFC. Reduced resection of the vertebral lamina preserves the structure of the posterior column, without the increased difficulty in the full exposure of the spinal canal. Following accurate fenestration, full undercutting decompression and dealing with the basal part of the spinous process, the spinal canal may be well-exposed. Following exploration of the spinal canal and confirmation that the dural sac and nerve root have been relieved from compression and fully loosened, the neural hook and TFC protection devices may be properly placed. The C-arm X-ray machine is used to monitor and distinguish the direction and depth of the intervertebral disc space. According to pre-operative assessment, a TFC with appropriate length and diameter is selected to quickly and efficiently complete the vertebral fusion with its self-drilling screw structure (23). iv) Adhesion around the spinal canal should be actively prevented. Ice-cold NS is used intra-operatively for flushing, which reduces hemorrhage and reactive edema, as well as cicatrix in the spinal canal. While closing the spinal canal, poly(d,l-lactic acid) is conventionally used to absorb the medical film and close the bone window. This material accords with the requirement to avoid epidural adhesion (24), without damaging or stimulating tissues. This material has a microporous structure and good adhesion and does not need to be sutured and fixed; therefore, it is maintained in the body for ∼2 months and completely absorbed and degraded within 6 months. The final degradation products are water and carbon dioxide. Therefore, this material is used to close the bone window as a membranous barrier and reduce the mechanical stimulus of cicatrix outside the spinal canal and adhesion to the dural sac.

In summary, the final purpose in the treatment of lower lumbar spinal instability was lumbar spinal osseous fusion and long-term stability. Clinical study has indicated that the PLIF technology demonstrates a better accordance with the natural biomechanical features of the lumbar spine (25). If bilateral decompression via vertebral lamina fenestration is performed, the principle of vertebral pressure support and the tension band principle of posterior structures (26) is effectively protected. This is ideal to achieve a steady vertebral interbody fusion and effective spinal decompression, which is significant for developing a lumbar spinal fusion procedure that is effective, minimally invasive and normalizing at a reduced cost. Further studies with numerous centers and related multiple factor analysis, including ethnicity, physique, pathogenesis and spinal canal forms, would aid the treatment of lower lumbar spinal instability by combining the ‘limited surgical approach’ with effective decompression and fusion.
